# Supplementary material for: Salivary interleukin-17A and interleukin-18 levels in patients with celiac disease and periodontitis
Source: PeerJ. 2024 May 13;12:e17374. doi: 10.7717/peerj.17374 (PMC11097963; doi:10.7717/peerj.17374)
Supplement: Supplemental Information 1 [file peerj-12-17374-s001.docx]

**Gender:**

1-Male
2-Female

**Periodontal condition:**

0-non-periodontitis
1-Periodontitis

**Endomycial (EMA):**

0- not done
1-positive
2-negative Or weak positive
3- Positive in re-test

**Anti_gliadin_Ab**

0- not done
1-positive
2-negative Or weak positive
3- Positive in re-test

**Anti-tTG (IgA)**

0- not done
1-positive
2-negative Or weak positive
3- Positive in re-test

**Anti-tTG IgG**

0- not done
1-positive
2-negative Or weak positive
3- Positive in re-test
